# Combined Influence of Visual Scene and Body Tilt on Arm Pointing Movements: Gravity Matters!

**DOI:** 10.1371/journal.pone.0099866

**Published:** 2014-06-12

**Authors:** Cécile Scotto Di Cesare, Fabrice R. Sarlegna, Christophe Bourdin, Daniel R. Mestre, Lionel Bringoux

**Affiliations:** Aix-Marseille Université, CNRS, ISM UMR 7287, Marseille, France; VU University Amsterdam, Netherlands

## Abstract

Performing accurate actions such as goal-directed arm movements requires taking into account visual and body orientation cues to localize the target in space and produce appropriate reaching motor commands. We experimentally tilted the body and/or the visual scene to investigate how visual and body orientation cues are combined for the control of unseen arm movements. Subjects were asked to point toward a visual target using an upward movement during slow body and/or visual scene tilts. When the scene was tilted, final pointing errors varied as a function of the direction of the scene tilt (forward or backward). Actual forward body tilt resulted in systematic target undershoots, suggesting that the brain may have overcompensated for the biomechanical movement facilitation arising from body tilt. Combined body and visual scene tilts also affected final pointing errors according to the orientation of the visual scene. The data were further analysed using either a body-centered or a gravity-centered reference frame to encode visual scene orientation with simple additive models (i.e., ‘combined’ tilts equal to the sum of ‘single’ tilts). We found that the body-centered model could account only for some of the data regarding kinematic parameters and final errors. In contrast, the gravity-centered modeling in which the body and visual scene orientations were referred to vertical could explain all of these data. Therefore, our findings suggest that the brain uses gravity, thanks to its invariant properties, as a reference for the combination of visual and non-visual cues.

## Introduction

The brain continuously receives a flow of spatial information from several sensory channels about the ever-changing states of the environment and the body. Producing an appropriate behaviour such as goal-directed arm movements involves continuous adjustments in response to, for instance, active or passive body displacements. Indeed, when pointing toward an object while being tilted, the Central Nervous System (CNS) has to take into account the directional shift of gravitational force which is no longer aligned with the longitudinal body axis. In addition to force-field characteristics, visual cues due to body or object displacement are also integrated. This is illustrated by the fact that a tilt of the visual scene influences the perceived orientation of the self or of an object to be reached [1], [2]. Here we investigated the influence of body and/or visual scene tilts on arm pointing movements to better understand the processes underlying body and target localization as well as motor planning and control. This study specifically focused on the combination of spatial cues at the basis of sensorimotor control during combined body and visual scene tilts.

Tilting the visual scene has been found to influence many spatial orientation tasks such as the judgment of visual straight ahead or longitudinal head axis [3], [4]. In addition to these perceptual judgments, motor consequences of visual scene tilts have also been reported on arm pointing movements [1,2,5: unpublished]. For instance, Welch and Post [1] showed that the final accuracy of reaching movements was altered as a function of the direction of the visual scene tilt in pitch. These authors argued that final errors were mainly due to the inability to accurately localize the physical eye level. Subjective eye level has indeed been shown to be linearly influenced by the pitch tilt of the visual scene [4], [6], [7]. Since target position in elevation, even referred to a body-fixed reference, has been found to be partly coded relative to eye level [8], [9], perceived target location would be consequently impaired when the visual scene is tilted. These previous studies [1], [2], [5] exclusively focused on the effects of visual scene pitch tilt on final accuracy and not on motor organization. However, a detailed kinematic analysis is required to finely understand sensorimotor control processes.

Contrasting with visual scene tilt, tilting the body in roll or in pitch has biomechanical consequences as the gravitational vector is no longer aligned with the longitudinal body axis. The CNS must then update the gravity-related constraints applied to the body, and particularly to the arm, for maintaining the accuracy of goal-directed movements. However, the few studies dealing with the influence of pitch body tilt on arm pointing movements presented contradictory results [2], [10], [11]. While Smetanin and Popov [11] found target overshoots associated to upward pointing movements during prone or supine body orientation, other studies did not show any significant influence of fast (12 deg.s^−1^, [2]) or slow pitch body tilt (0.05 deg.s^−1^, [10]) on final pointing accuracy. Analyzing arm movement kinematics may help further understand these seemingly contradictory results. For instance, Le Seac'h and McIntyre [12] reported that the timing and shape of arm pointing movement varied relative to body orientation in the roll dimension (i.e., vertical posture vs. reclined on the left side) which may account for changes in final position. Here, we analysed movement kinematics to determine how well subjects predicted the consequences of gravity on the arm and whether they adjusted their movement during its execution.

The core issue of the present study concerned the way spatial cues relative to visual scene and body orientation are combined for the planning and control of a goal-directed arm movement. It is well established that combined body and visual scene tilts influence the judgement of body orientation [13]–[15]. However, while some studies revealed that judgement errors during combined body and scene tilts mainly corresponded to visual errors [16], [17], other studies showed that errors during combined head and visual scene tilts appeared as an additive combination of the errors observed during each single tilt [18]. To our knowledge, only Fouque et al. [2] investigated the influence of combined body and visual scene tilts in pitch on sensorimotor control. These authors showed that the accuracy of pointing movements toward a visual target presented at eye level could be impaired when coupling body and scene tilts arising from fast rotations. Final errors were similar to those observed during visual scene tilt alone as body tilt alone did not seem to affect movement endpoint. In this work [2], the fact that body tilt had no significant influence on final accuracy may be due to the correct compensation of gravity action on the body [19]–[22], which may have been facilitated by the fast (i.e., v = 12 deg.s^−1^), easily detectable rotation pattern. It is unclear, however, what may happen in the case of tilts below semi-circular canals thresholds [23], [24] which may complexify the compensation of gravity constraints on the body.

Here, we tested the influence of very slow (i.e., 0.05 deg.s^−1^) body and/or visual tilts on the final accuracy of arm pointing movements which reflects motor planning and online control mechanisms [25], [26] and we also analysed early kinematic parameters which mostly reflect motor planning [27], [28]. Combined conditions were manipulated so that the direction of body and visual scene tilts remained unchanged or was shifted. This gave us the opportunity to study the combination process of spatial cues underlying the control of arm pointing movements. Specifically, we tested whether the subjects' motor behavior better corresponds to an egocentric (i.e., body-centered) or external (i.e., gravity-centered) encoding of sensory information.

## Methods

### Participants

Fifteen right-handed subjects (9 men and 6 women; mean age ± SD: 23±3 years) were recruited from the students and staff of Aix-Marseille University to participate in this experiment. Right hand preference was assessed with the 10-item version of the Edinburgh handedness inventory [29], and all subjects had a laterality quotient greater than 50. Subjects reported having normal or corrected-to-normal vision and no neurological or sensorimotor disorders. Stereoscopic vision was checked using the Randot Stereotest® with all individual scores greater than 70 s of arc. All participants gave written informed consent prior to the study, in accordance with the 1964 Declaration of Helsinki and the written consent of a local institutional review board (IRB) from the Institute of Movement Sciences which specifically approved this study.

### Apparatus

**Figure 1:**
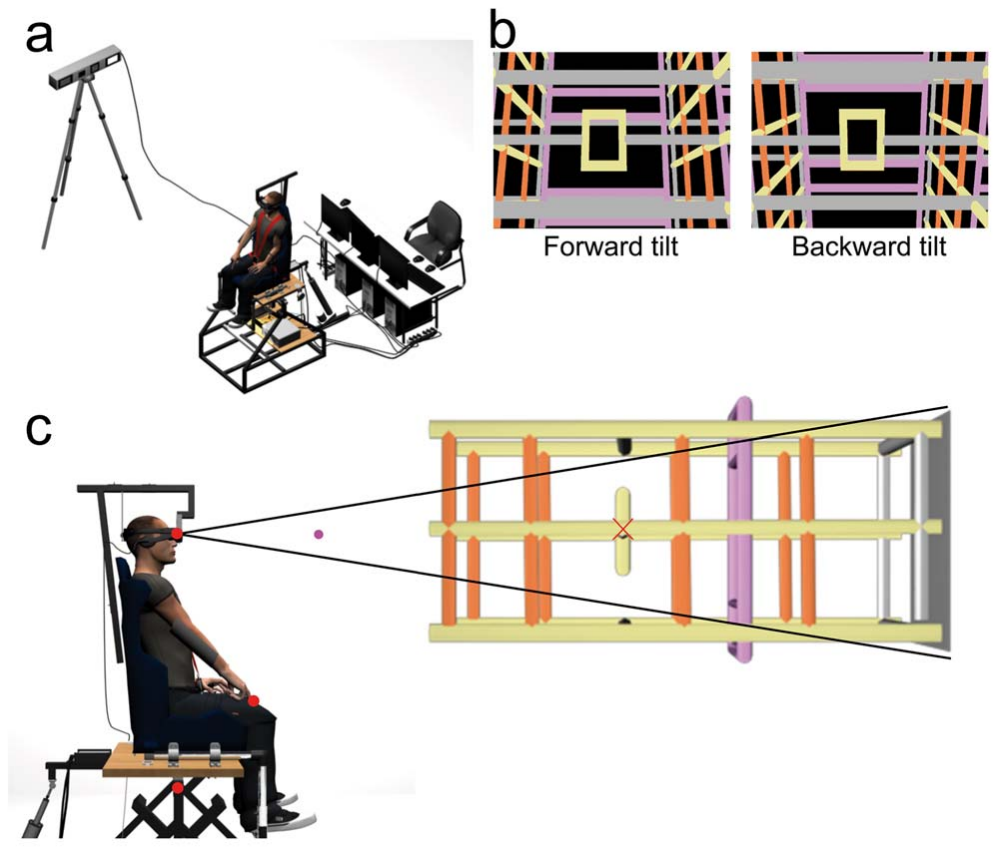
Experimental setup. **a**) Global view of the apparatus including the tilting chair, the HMD, the motion tracking system and the cluster of computers. **b**) Side view of the tilting chair. The sketch represents a subject in the initial standard position with the right arm outstretched in a drainpipe. Red dots represent the markers tracked with the motion capture system. They were positioned on the index fingertip, at eye level, and on the chair axis of rotation. The HMD displayed a visual target (pink dot) located straight ahead and a structured visual background as illustrated in front of the subject. The red cross, which was not displayed to subjects, corresponds to the center of scene rotation. **c**) Screen captures of the visual scene actually viewed by subjects' right eye when tilted 18 deg forward (left panel) and backward (right panel).

Subjects were seated on a tilting chair, firmly maintained by a six-point seatbelt (Fig. 1a). The chair could be tilted in the pitch dimension by rotating around an axis positioned under the seat (Fig. 1c). The chair was rotated by lengthening/shortening an electric jack (Phoenix Mecano, thrust: 3 kN, clearance: 0.6 m, precision 0.12 mm) attached to the back of the seat. The tilt angular profile was servo-assisted using an inclinometer fixed to the chair (AccuStar, resolution: 0.1 deg; range: ±60 deg). The tilt velocity was set at 0.05 deg.s^−1^ following an acceleration phase at 0.005 deg.s^−2^. An adjustable drainpipe was used to support the arm weight in the starting position to prevent arm fatigue. During the experimental trials, earphones provided white noise to mask any auditory cues (e.g., from the rotating chair or the computers).

A 3D head-mounted display (HMD, 3D Cybermind hi-Res900, Cybermind Interactive Nederland, The Netherlands; resolution: 800×600 pixels; field of view: 31.2 deg diagonal for each eye) was fixed horizontally onto a headrest attached to the seat. This headrest was adjustable in elevation to the subject size. The HMD was used to display a stereoscopic visual background. The visual scene was composed of a 3D grid that reinforced horizontal and vertical reference lines positioned at different depth levels (overall scene depth: 3.15 m, see Figure 1b and c). The front of the scene was positioned at 1.5 m from eye position. The scene could rotate in the pitch dimension, around an axis of rotation positioned at 2.65 m from eye position (i.e., 1.15 m further from the visual scene front) in the middle of the screen in the vertical plane (Fig. 1b). Because rotating the scene around the chair axis of rotation might induce several additional illusions due to vertical translational optic flow (e.g., target induced motion, [30] and vection, [31]), which could induce opposite effects on arm pointing movements, we rotated the background around the centre of the screen to minimize the occurrence of such illusions. This specific rotation is sufficient to induce errors in judgement relative to the environment or to the body as simple tilted planes did [32], [33]. A pink virtual target (diameter: 1 cm) was projected at the centre of this visual background, in the frontal plane and was always fixed relative to the observer, even during visual and/or body tilts; i.e., the target was positioned at Head-Referenced Eye Level (HREL: transversal plane of the head passing through the eyes; [34]). The target was presented at 0.8 m from the eye position. The HMD device prevented subjects from having visual feedback about the experimental setup and about their current arm location.

An optical motion tracking system (Codamotion cx1 and MiniHub, Charnwood Dynamics Ltd, Leicestershire, UK), was placed at 2.5 m laterally from the chair and 1.9 m vertically from the ground. Infrared active markers were placed on the right index fingertip and at eye level on the HMD to compute angular pointing errors. Markers' position data were sampled at 200 Hz. A real-time acquisition system (ADwin-Pro, Jäger, Lorsch, Germany) running at 10 kHz was driven by a customized software (Docometre) to synchronously control kinematic data collection as well as visual background and/or chair tilts.

### Procedure

During the experiment, subjects sat on the rotating chair and were prompted to point toward the visual target. For each condition, the chair and the visual background were initially set at 0 deg (i.e., at vertical). At the beginning of each pointing trial, subjects put their extended arm in the drainpipe and positioned their right index finger at the starting position, indicated by a standardised tactile landmark (a 2 cm^2^ piece of Velcro) on their right thigh. A double auditory signal announced the onset of a pointing block. Three seconds later, the visual target appeared with an auditory signal prompting the subjects to point toward the target. Subjects were asked to reach the target, which remained visible during 1 s, in a single-joint shoulder movement (arm outstretched) and to maintain final arm position until target disappearance. The task instructions were given as follows: ‘Once the target appears, reach the target with the arm outstretched, as fast and as accurately as possible. Target appearance is associated to an auditory tone. You have to reach the target before its disappearance. When the target is extinguished, bring your outstretched arm back to the standard position'. This standard position corresponded to the arm in the drainpipe and the index finger on the tactile landmark. A new target appeared 3 s after the previous target disappeared. This sequence was repeated 6 times and constituted a pointing block.

**Figure 2:**
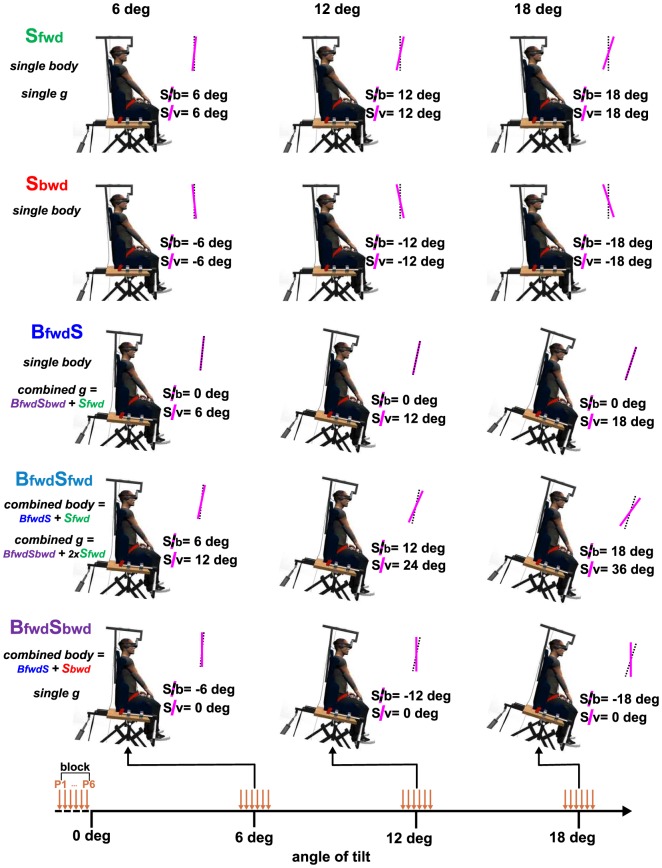
Experimental conditions and procedure. Body and/or visual scene tilts are depicted for angles at which pointing movements were requested (i.e., 6, 12 and 18 deg) for each experimental condition (**S_fwd_**, **S_bwd_**, **B_fwd_S**, **B_fwd_S_bwd_**, **B_fwd_S_fwd_**). Pink lines correspond to the visual scene orientations and dotted lines to the longitudinal body orientations. We mentioned the angle of visual scene orientation relative to the longitudinal body orientation (i.e., in a body-centered reference frame) as ‘S/b’ and relative to vertical as ‘S/v’ (i.e., in a gravity-centered reference frame). Associated single and combined conditions relative to the body-centered (i.e., body) and gravity-centered (i.e., g) reference frame are provided under each experimental condition. The lower panel of the figure illustrates the sequence of events including the different pointing blocks required during a trial (i.e., from 0 to 18 deg of body and/or visual scene tilt relative to the observer).

The sequence of events for each experimental condition is illustrated in Figure 2. Pointing blocks were performed at 0, 6, 12 and 18 deg during continuous body and/or visual tilts from 0 to 19 deg. The rotating chair was not stopped during the pointing blocks to avoid any effect of acceleration and deceleration phases. Since the same spatiotemporal profiles were used for visual and body tilts, the tilt of the visual scene was not stopped during pointing blocks. As a consequence, a pointing block was designed to start 0.5 deg before the intended angle of body and/or visual tilts and to end 0.5 deg after. For example, to assess the effect of a 6 deg body or visual scene tilt, arm pointing movements were performed each 0.2 deg from 5.5 deg to 6.5 deg of tilt.

Subjects were also required to verbally indicate whether they felt tilted when prompted by an auditory tone differing from that used in the pointing task. This perceptual task was repeated every 2 deg, from 1 deg to 19 deg of body and/or visual scene tilts. Results related to this concurrent task will be presented in details elsewhere.

Once the body and/or the visual scene were tilted by 19 deg, the visual scene disappeared. If the body was actually tilted, the chair was tilted back to 0 deg with a pseudo-random profile in which we varied the magnitude and duration of the acceleration and deceleration phases. Between conditions, the HMD was removed and a period of rest in full ambient light during at least 1 min was consistently provided before the next condition started. This resting period was used to suppress post-rotational effects due to semi-circular canal stimulation [23], [24] and to limit possible fatigue. The subsequent body and/or visual scene tilts condition began only when subjects did not feel tilted anymore.

The experimental conditions consisted in tilts of the body and/or the visual scene in the pitch dimension with forward tilts (body and/or visual scene) and backward tilt (visual scene only) up to 19 deg using the same velocity profile. We chose to perform only forward body tilt as we expected that this direction would yield larger consequences on arm pointing movement. Indeed, the results of Fouque et al. [2] showed no significant errors for fast backward body tilt while a trend could be observed for forward body tilt. Figure 2 illustrates the 5 experimental conditions tested in the present study: **S_fwd_**: forward visual scene tilt (top of the visual scene away from the observer) without body tilt; **S_bwd_**: backward visual scene tilt (top toward the observer) without body tilt; **B_fwd_S**: forward body tilt with a visual **s**cene kept parallel relative to the subject; **B_fwd_S_fwd:_** forward body tilt and forward visual scene tilt; **B_fwd_S_bwd:_** forward body tilt with backward visual scene tilt. These experimental conditions and their associated names were defined in a body-centered reference frame (i.e., visual scene referred to the observer, Fig. 2).

All 15 subjects performed 3 repetitions in each of the 5 aforementioned conditions, which were presented in a pseudo-random, counterbalanced order. A training session without body and/or visual scene tilts was provided before data collection actually started to familiarize subjects with both perceptual and motor tasks. The whole experimental session lasted about 2 hours.

### Data processing

Position data from the markers on the right index fingertip, the HMD and the rotation axis of the rotating chair were low-pass filtered with a dual-pass, no-lag Butterworth filter (cut-off frequency: 10 Hz; order: 2). This allowed us to compute the angular pointing position in the sagittal plane relative to the eye elevation (i.e., HREL) for the entire movement, which took into account instantaneous chair orientation. Arm movement onset and offset were defined when angular velocity in the sagittal plane respectively reached above and dropped below 5% of peak velocity [10], [19], [21], [27], [35], [36]. Final position (i.e., movement endpoint) was calculated from the angle between the index and HREL (i.e., target location). Selected kinematic parameters were peak acceleration (PA), time-to-peak acceleration relative to movement duration (rTPA), reaction time (RT) and movement duration (MD).

The main purpose of the subsequent analyses was to test the effect of tilt in the different experimental conditions. Prior to this issue, we investigated any potential order effect of the 6 successive arm pointing movements composing a pointing block. To that aim, we conducted 5 condition (**S_fwd_**, **S_bwd_**, **B_fwd_S**, **B_fwd_S_fwd_**, **B_fwd_S_bwd_**)×4 angle (0, 6, 12, 18 deg)×6 pointing succession (number 1 to number 6) repeated-measures ANOVAs on the mean of the 3 repetitions for all kinematic parameters. These analyses did not reveal any significant effect of pointing succession upon the interaction condition x angle of tilt, and statistical comparisons were conducted on the mean (i.e., average of the 6 pointing movements ×3 repetitions) for all experimental conditions and angles of tilt, using 5 condition (**S_fwd_**, **S_bwd_**, **B_fwd_S**, **B_fwd_S_fwd_**, **B_fwd_S_bwd_**) ×4 angle of tilt (0, 6, 12, 18 deg) repeated-measures ANOVAs. As we wanted to focus on the general effect of tilt, planned comparisons were systematically performed to contrast the control situation at 0 deg vs. all the tilted situations (6 deg, 12 deg and 18 deg).

#### Modeling

First, we considered ‘single’ vs. ‘combined’ conditions when coding visual scene orientation in a body-centered reference frame (see Figure 2). Using these spatial coordinates, **S_fwd_**, **S_bwd_**, and **B_fwd_S** corresponded to single conditions (i.e., single rotation of the body or the visual scene relative to the observer), and **B_fwd_S_fwd_** and **B_fwd_S_bwd_** to combined conditions (i.e., combined rotations of the body and the visual scene relative to the observer). We thus examined whether kinematic parameters observed in these combined conditions could correspond to the additive combination (i.e., unweighted sum) of the data observed in the corresponding single conditions. To that aim, we rebased data relative to final position, PA, rTPA, RT and MD so that this unweighted sum model could be applicable. Hence, for a given data type, the mean of a given parameter obtained at 0 deg was subtracted from the means obtained for tilted orientations. Data predicted by this model were computed by simply adding the mean values (average of the 6 pointing movements x 3 repetitions) issued from each single condition associated to both combined condition for each kinematic parameter (final position, PA, rTPA, RT and MD). Hence, for each subject, **B_fwd_S_fwd_** predicted data corresponded to the unweighted algebraic sum of **B_fwd_S** and **S_fwd_** data, and **B_fwd_S_bwd_** predicted data corresponded to the unweighted algebraic sum of **B_fwd_S** and **S_bwd_** data. We then tested whether this unweighted sum model could predict the combined conditions for a given kinematic parameter using 2 data type (observed data from a combined condition vs_._ predicted data from body-centered modeling) x 3 angle of tilt (6, 12, 18 deg) repeated-measures ANOVAs for each combined condition (**B_fwd_S_bwd_**, **B_fwd_S_bwd_**). Note that 0 deg was excluded from the analyses because the mean of both data types at this angle was always set at 0 for all parameters. In order to compare observed and predicted data, we focused our interest on the comparison between data types as well as from the interaction data type x angle of tilt.

Alternatively, we considered ‘single’ vs. ‘combined’ conditions when coding visual scene and body orientation in a gravity-centered reference frame (see Figure 2). Using these spatial coordinates, **S_fwd_**, and **B_fwd_S_bwd_** were defined as single conditions (i.e., single rotation of the body or the visual scene relative to vertical), and **B_fwd_S** and **B_fwd_S_fwd_** as combined conditions (i.e., combined rotations of the body and the visual scene relative to vertical). With this gravity-centered modeling, predicted data **B_fwd_S** corresponded to the algebraic sum of **B_fwd_S_bwd_** and **S_fwd_** data, and **B_fwd_S_fwd_** predicted data corresponded to the algebraic sum: **B_fwd_S_bwd_**+2×**S_fwd_**. As previously, data predicted by this model were computed for each rebased kinematic parameter (final position, PA, rTPA, RT and MD). We also tested whether this gravity-centered model could predict these new combined conditions for a given variable using a 2 data type (observed data from a combined condition vs_._ predicted data from gravity-centered modeling) ×3 angle of tilt (6, 12, 18 deg) repeated-measures ANOVAs.

Overall, post-hoc tests (Newman-Keuls) were performed when necessary and the level of significance was set at .05 for all statistical analyses.

## Results

Prior analyses were conducted to test any potential effect of the 6 successive pointing movements within each pointing block that could interact with the factors manipulated in the study. The analyses, detailed in the *Appendix*, revealed that even though an effect of pointing succession or an interaction between this factor and the angle of tilt appeared for several parameters; i) it cannot be considered as a consequence of the experimental design itself (i.e., continuous rotation); ii) it did not influence the interaction condition x angle of tilt for any given variable, this interaction representing the core interest of the study. Therefore, for the sake of clarity, we averaged in the subsequent analyses the values obtained from 6 pointing movements ×3 repetitions, hence yielding a mean individual observation for a given condition at a given tilt. The first part of the result section reports behavioural data while the second section is dedicated to modeling.

### Final pointing accuracy

The analysis of final accuracy revealed an effect of condition (F_(4,56)_ = 4.4; p<.01), angle of tilt (F_(3,42)_ = 6.7; p<.001) as well as an interaction condition x angle of tilt (F_(12,168)_ = 7.2; p<.001). It should be noted that none of the post-hoc results revealed a difference between conditions at 0 deg, indicating that the baseline was similar in all experimental conditions. The subsequent description focused on comparisons between angles of tilt (i.e., 0, 6, 12 and 18 deg) for a given condition, and notably on positions relative to the baseline of 0 deg (i.e., final errors).

**Figure 3:**
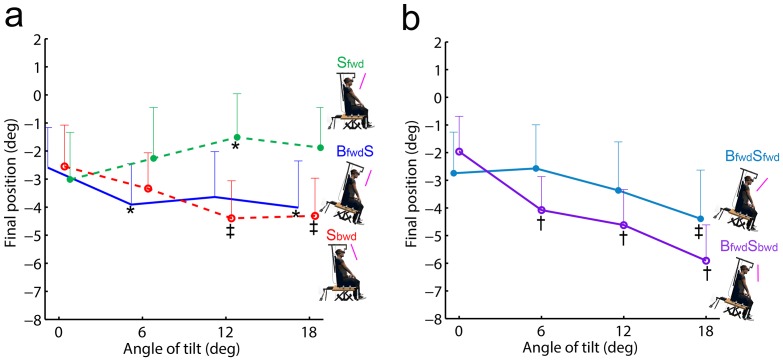
Final pointing position as a function of conditions and angles of tilt. **a**) **Single conditions (S_fwd,_ S_bwd_ and B_fwd_S) relative to the angle of tilt.** **b**) Combined conditions (**B_fwd_S_bwd_** and **B_fwd_S_fwd_**) relative to the angle of tilt. Symbol positioned below a given value of a specific angle and condition represents a statistical difference with 0 deg in this specific condition (*: p<.05; ‡: p<.01; †: p<.001). Note that final positions are also statistically different for **B_fwd_S_bwd_** between 6 deg vs. 12 deg or 18 deg (p<.001 and p<.01, respectively) and for **B_fwd_S_fwd_** between 6 deg vs. 18 deg (p<.01). Conditions are illustrated on the right side of each figure with pink lines representing the scene orientation (N.B., scene depth distance was not at scale). Vertical bars denote positive standard errors.

Overall, the angle of tilt influenced final pointing accuracy for each of the single conditions (**S_fwd_**_,_ **S_bwd_** and **B_fwd_S**, Fig. 3a). Positive positions relative to baseline (overshoots) were found in **S_fwd_** and negative positions relative to baseline (undershoots) in **S_bwd_** with errors increasing from 0 to 12 deg and remaining stable between 12 and 18 deg. Body tilt without scene tilt (**B_fwd_S**) induced negative errors with approximately the same magnitude at 6, 12 deg and 18 deg (difference of −1.3±1.5 deg., −1.1±1.7 deg and −1.4±1.7 deg relative to the baseline, respectively; no statistical difference between angles of tilt). Combined body tilt with scene tilted forward (**B_fwd_S_fwd_**) or scene tilted backward (**B_fwd_S_bwd_**) both yielded negative errors relative to baseline (Fig. 3b). However, while errors in **B_fwd_S_fwd_** differed from baseline only at 18 deg (−1.7±1.8 deg of difference; p<.01), errors in **B_fwd_S_bwd_** differed from baseline at each angle of tilt (−2.1±1.2 deg at 6 deg, −2.7±1.3 deg at 12 deg and −3.9±1.3 deg at 18 deg of difference; p<.001 for all comparisons). In addition, it should be noted that final position in **B_fwd_S_fwd_** differed from that in **S_fwd_** at 12 and 18 deg (p<.01 and p<.001, respectively). By contrast, at 18 deg, positions in **B_fwd_S_bwd_** differed from positions in both **B_fwd_S** (p<.001) and **S_bwd_** (p<.01).

In order to focus on the overall effect of tilt upon conditions, whatever its magnitude, we performed planned comparisons between 0 deg vs. tilted situations (i.e., 6, 12 and 18 deg) for each condition. Planned comparisons showed statistical differences between errors in 0 deg and tilted situations for **S_bwd_** (p<.05), **B_fwd_S** (p<.05), **B_fwd_S_bwd_** (p<.001) but not for **S_fwd_** (p = .07) and **B_fwd_S_fwd_** (p = .28). This analysis thus confirmed the effect of tilt on the final pointing errors in **S_bwd_**, **B_fwd_S** and **B_fwd_S_bwd_** conditions regardless of the tilt magnitude.

### Movement kinematics (PA, rTPA, RT and MD)

**Figure 4:**
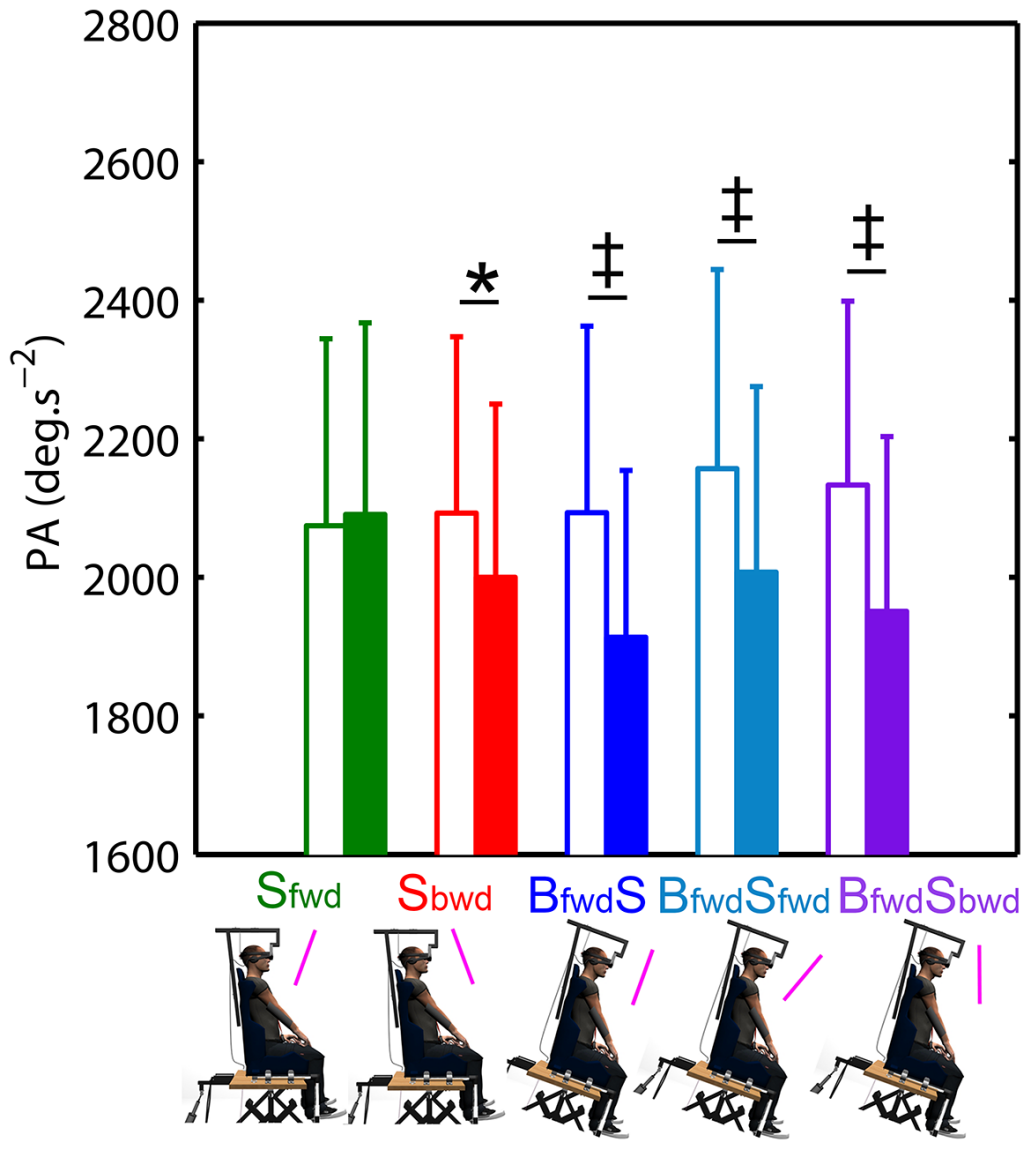
Peak acceleration (PA) as a function of condition and body and/or visual scene orientation (0 deg: white bars; tilted: coloured bars). Differences in planned comparisons between tilted vs. 0*: p<.05; ‡: p<.01; †: p<.001.

We analysed peak acceleration (PA) to assess whether, in parallel to final accuracy, an early modification of movement pattern also appeared as a function of angle of tilt and condition. The 5 condition ×4 angle of tilt repeated-measures ANOVA revealed a significant effect of condition (F_(4,56)_ = 2.7; p<.05), angle of tilt (F_(3,42)_ = 7.7; p<.001) as well as a significant interaction condition x angle of tilt (F_(12,168)_ = 3.2; p<.001). Figure 4, which depicts the results of planned comparisons for PA as a function of condition and orientation (0 deg vs. tilted), shows that PA was smaller when the body was actually tilted, whatever the scene orientation, as compared to 0 deg body orientation (mean difference of 180±46 deg.s^−2^, p<.01; 150±48 m.s^−2^, p<.01 and 182±45 deg.s^−2^, p<.01; in **B_fwd_S**, **B_fwd_S_fwd_** and **B_fwd_S_bwd_** conditions, respectively). In the **S_bwd_** condition in which final undershoots were observed, a smaller PA was also found when tilted as compared to the 0 deg body orientation (mean difference: 92±42 deg.s^−2^, p<.05).

**Figure 5:**
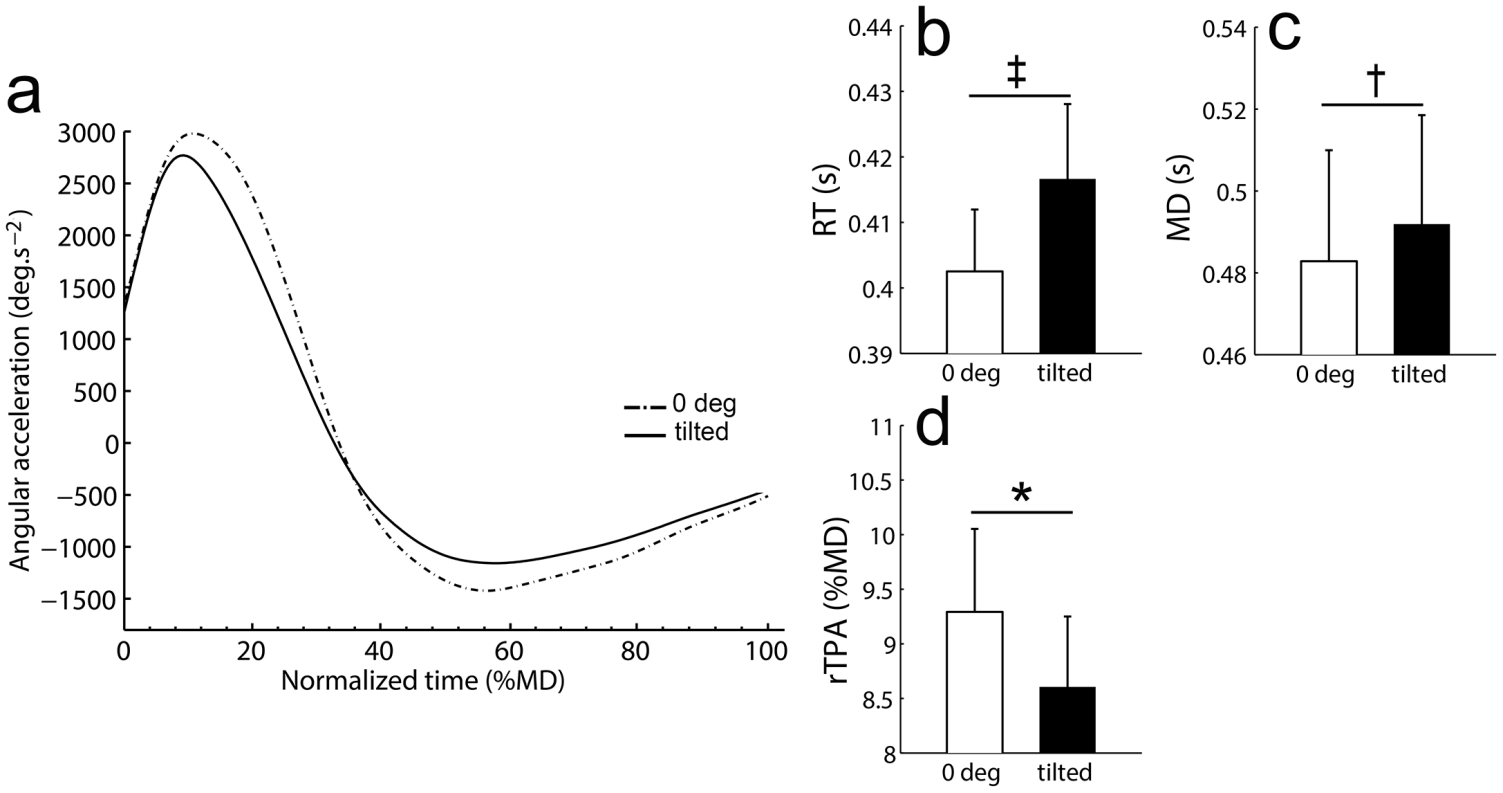
Movement pattern relative to orientation (0 deg vs. tilted). **a**) Typical normalized acceleration profile relative to MD as a function of orientation (mean of all conditions). Differences in planned comparisons between tilted vs. 0 deg orientations were provided on the right panel for rTPA (**b**), RT (**c**), and MD (**d**). Vertical bars denote positive standard errors. *: p<.05; ‡: p<.01; †: p<.001.

Figure 5a shows that some common spatiotemporal features of the movement were observed when the body and/or the scene was tilted as compared to 0 deg orientation. This was confirmed by the presence of a main effect of the angle of tilt for rTPA (F_(3,42)_ = 5.8; p<.01), RT (F_(3,42)_ = 10.1; p<.001) and MD (F_(3,42)_ = 5.3; p<.01) revealed by 5 condition ×4 angle of tilt repeated-measures ANOVAs. In addition, there was neither main effect of condition (rTPA: F_(4,56)_ = 0.3; p = .85; RT: F_(4,56)_ = 0.5; p = .74, MD: F_(4,56)_ = 2.4; p = .06) nor interaction condition x angle of tilt (rTPA: F_(12,168)_ = 1.3; p = .20; RT: F_(12,168)_ = 0.9; p = .51, MD: F_(12,168)_ = 1.3; p = .24) for these parameters. Planned comparisons, thus based on the set of all experimental conditions, revealed a shorter time-to-peak acceleration relative to MD (rTPA) in tilted situations as compared to 0 deg (8.6±0.7%MD vs. 9.3±0.8%MD; Fig. 5b). Second, RT in tilted situations was higher as compared to 0 deg (417±12 ms vs. 403±10 ms; Fig. 5c). Third, MD was longer in tilted situations as compared to 0 deg (492±28 ms vs. 483±28 ms; Fig. 5d).

In summary, both common (rTPA, RT and MD) and condition-specific (PA) changes were found on arm kinematics when the body and/or the scene was tilted as compared to vertical orientation.

### Body-centered modeling

We first examined whether the previous variables (final position, PA, rTPA, RT, MD) observed in the combined conditions defined in a body-centered reference frame (i.e., **B_fwd_S_fwd_** and **B_fwd_S_bwd_**) could be predicted by the unweighted sum of associated single conditions (**S_fwd_**_,_ **S_bwd_** and **B_fwd_S**).

**Figure 6:**
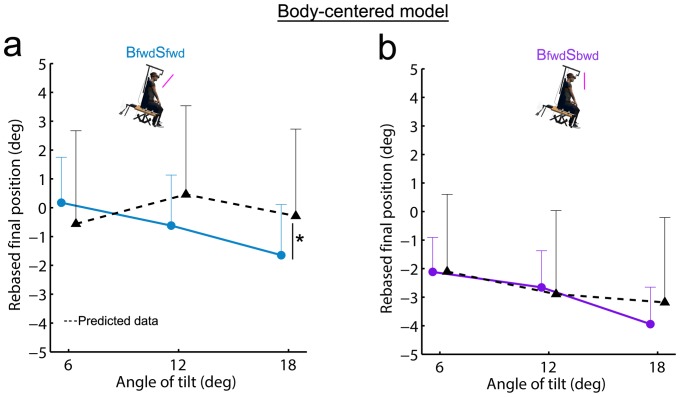
Final pointing position observed in combined conditions (solid lines) and associated predicted data (black dotted line) by the body-centered model. **a**) Combined condition **B_fwd_S_fwd_** and predicted data by the unweighted sum. **b**) Combined condition **B_fwd_S_bwd_** and predicted data by this unweighted sum. Vertical bars denote positive standard errors. *: p<.05; ‡: p<.01; †: p<.001.

We first compared observed and predicted data regarding final pointing errors in **B_fwd_S_fwd_** on the one hand and in **B_fwd_S_bwd_** on the other hand. In **B_fwd_S_fwd_**, the repeated-measures ANOVA revealed no main effect of data type (F_(1,14)_ = 0.1; p = .72) nor angle of tilt (F_(2,28)_ = 2.2; p = .13) but showed an interaction data type x angle of tilt (F_(2,28)_ = 3.4; p<.01). At 6 and 12 deg, the data observed in **B_fwd_S_fwd_** did not differ from the data predicted by this unweighted sum model (Fig. 6a). At 18 deg however, the predicted data statistically differed from the observed data (p<.05).

In **B_fwd_S_bwd_**, the repeated-measures ANOVA revealed a main effect of the angle of tilt (F_(2,28)_ = 5.1; p<.05). However, the analysis did not reveal any significant effect of data type (F_(1,14)_ = 0.0; p = .92) nor interaction data type x angle of tilt (F_(2,28)_ = 1.4; p = .24), indicating that data predicted by this unweighted sum did not differ from the observed data at all angles.

We used similar analyses on PA, rTPA, RT and MD to determine whether the observed data in combined conditions could be determined by this body-centered unweighted sum model.

**Figure 7:**
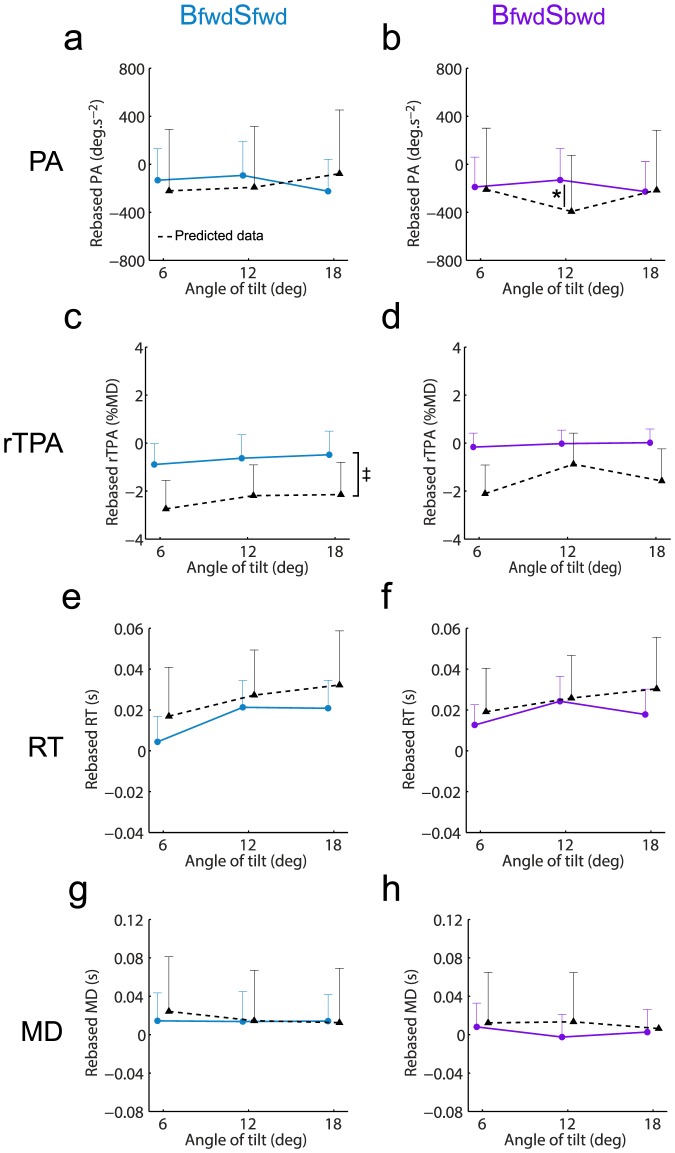
Kinematic parameters observed in combined conditions (solid lines) and associated predicted data (black dotted line) by the body-centered model. Observed and predicted data for PA (**a,b**), rTPA (**c,d**), RT (**e,f**) and MD (**g,h**) were provided for both combined conditions (left panel: **B_fwd_S_fwd_**; right panel **B_fwd_S_bwd_**). Vertical bars denote positive standard errors. The lines between conditions depict differences at a given angle and the bracket depicts an overall difference between conditions (i.e., effect of data type without interaction data type x angle of tilt). *: p<.05; ‡: p<.01; †: p<.001.

Regarding PA, the repeated-measures ANOVA performed for **B_fwd_S_fwd_** revealed no significant effect of the angle of tilt (F_(2,28)_ = 0.4; p = .70) nor data type (F_(1,14)_ = 0.0; p = .96). Even if we found an interaction data type x angle of tilt (F_(2,28)_ = 4.8; p<.05), post-hoc tests revealed that data predicted by this unweighted sum model did not statistically differ from the observed data (at 6 deg: p = .35; at 12 deg: p = .27; at 18 deg: p = .22; Fig. 7a). For **B_fwd_S_bwd_**, repeated-measures ANOVA also showed no effect of angle of tilt (F_(2,28)_ = 0.8; p = .48) nor data type (F_(1,14)_ = 0.1; p = .73). The interaction data type x angle of tilt (F_(2,28)_ = 4.0; p<.05) indicated that the predicted data differed from the observed data at 12 deg (p<.05; Fig. 7b). However, as observed data showed that PA was similar for most of the experimental conditions (see Figure 4), the results regarding PA modeling should be taken with caution.

Regarding rTPA, the repeated-measures ANOVA performed for **B_fwd_S_fwd_** showed a main effect of data type (F_(1,14)_ = 16.3; p<.01) but no effect of angle of tilt (F_(2,28)_ = 1.2; p = .30) nor interaction data type x angle of tilt (F_(2,28)_ = 0.1; p = .88). Overall, the predicted data thus differed from observed data (Fig. 7c). The analysis for **B_fwd_S_bwd_** revealed no main effect of data type (F_(1,14)_ = 2.5; p = .13), angle of tilt (F_(2,28)_ = 2.2; p = .13) nor interaction data type x angle of tilt (F_(2,28)_ = 1.1; p = .36; Fig. 7d).

Regarding RT, data predicted by this unweighted sum model did not differ from observed data for both **B_fwd_S_fwd_** and **B_fwd_S_bwd_** conditions (Fig. 7e and Fig. 7f) as attested by repeated-measures ANOVAs. No main effect of data type was found (**B_fwd_S_fwd_**: F_(1,14)_ = 0.7; p = .41; **B_fwd_S_bwd_**: F_(1,14)_ = 0.3; p = .61) nor interaction data type x angle of tilt (**B_fwd_S_fwd_**: F_(2,28)_ = 0.3; p = .75; **B_fwd_S_bwd_**: F_(2,28)_ = 0.5; p = .62). The analyses revealed an effect of angle of tilt for **B_fwd_S_fwd_** (F_(2,28)_ = 5.3; p<.05) but not for **B_fwd_S_bwd_** (F_(2,28)_ = 1.0; p = .36).

With respect to MD (Fig. 7g and 7h), repeated-measures ANOVAs neither revealed any main effect of data type (**B_fwd_S_fwd_**: F_(1,14)_ = 0.0; p = .92; **B_fwd_S_bwd_**: F_(1,14)_ = 0.1; p = .80), angle of tilt (**B_fwd_S_fwd_**: F_(2,28)_ = 0.9; p = .40; **B_fwd_S_bwd_**: F_(2,28)_ = 0.9; p = .40), nor interaction data type x angle of tilt (**B_fwd_S_fwd_**: F_(2,28)_ = 0.6; p = .56; **B_fwd_S_bwd_**: F_(2,28)_ = 1.4; p = .26).

Overall, considering this model in a body-centered reference frame, no clear conclusion could be drawn regarding the combination of visual and body orientation cues when looking at PA, rTPA, RT and MD. Regarding final accuracy, the model could account for the data in **B_fwd_S_fwd_** condition but failed to account for the data at each angle of tilt in **B_fwd_S_bwd_** condition. It is worth noticing, however, that the orientation of the visual scene relative to gravitational vertical differed in these combined conditions defined in a body-centered reference frame (i.e., in **B_fwd_S_bwd_** the scene is aligned with gravity whereas in **B_fwd_S_fwd_** the scene is tilted relative to gravity; see Figure 2). We then tested whether a similar model could be even more relevant only by reconsidering ‘single’ and ‘combined’ conditions in a gravity-centered reference frame.

### Gravity-centered modeling

**Figure 8:**
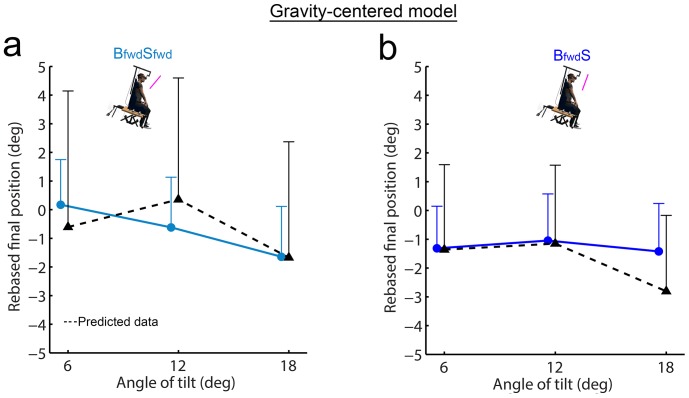
Final pointing position observed in the conditions B_fwd_S and B_fwd_S_fwd_ (solid lines) and associated predicted data (black dotted line) by the gravity-centered model. **a**) Combined condition **B_fwd_S_fwd_** and predicted data by this model. **b**) Combined condition **B_fwd_S** and predicted data by this model. Vertical bars denote positive standard errors.

We first compared observed and predicted data regarding final pointing positions for then new considered combined condition **B_fwd_S** on the one hand and **B_fwd_S_fwd_** on the other hand (Fig. 8). The repeated-measures ANOVA revealed a main effect of angle of tilt (**B_fwd_S**: F_(2,28)_ = 3.6; p<.05; **B_fwd_S_fwd_**: F_(2,28)_ = 3.4; p<.05) but no effect of data type (**B_fwd_S**: F_(1,14)_ = 0.2; p = .70; **B_fwd_S_fwd_**: F_(1,14)_ = 0.0; p = .98) nor interaction data type x angle of tilt (**B_fwd_S**: F_(2,28)_ = 2.0; p = .16; **B_fwd_S_fwd_**: F_(2,28)_ = 1.1; p = .36). Overall, these analyses indicated that final positions predicted by this gravity-centered model did not differ from observed data, whatever the angle of tilt.

**Figure 9:**
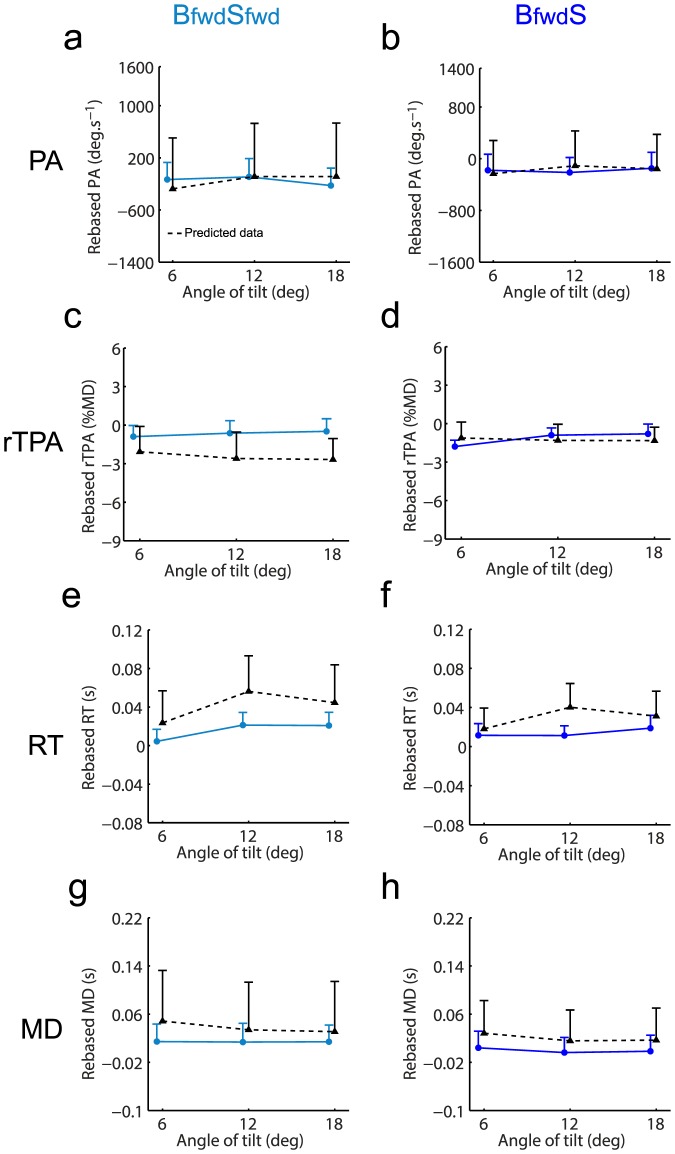
Kinematic parameters observed in conditions B_fwd_S and B_fwd_S_fwd_ (solid lines) and associated predicted data (black dotted line) by the gravity-centered model. Observed and predicted data for PA (**a,b**), rTPA (**c,d**), RT (**e,f**) and MD (**g,h**) were provided for both conditions (left panel: **B_fwd_S_fwd_**; right panel: **B_fwd_S**). Vertical bars denote positive standard errors. The lines between conditions depict differences at a given angle. *: p<.05.

We used similar analyses on PA, rTPA, RT and MD to determine whether the observed data in **B_fwd_S** and **B_fwd_S_fwd_** combined conditions would fit with the data issued from the gravity-centered modeling (Fig. 9).

Overall, we found no main effect of angle of tilt for PA (**B_fwd_S**: F_(2,28)_ = 0.9; p = .42; **B_fwd_S_fwd_**: F_(2,28)_ = 1.3; p = .29), rTPA (**B_fwd_S**: F_(2,28)_ = 0.9; p = .41; **B_fwd_S_fwd_**: F_(2,28)_ = 0.0; p = .97), RT (**B_fwd_S**: F_(2,28)_ = 2.0; p = .16) and MD (**B_fwd_S**: F_(2,28)_ = 2.9; p = .07; **B_fwd_S_fwd_**: F_(2,28)_ = 0.8; p = .48). A main effect of angle of tilt only appeared for RT in **B_fwd_S_fwd_** (F_(2,28)_ = 11.4; p<.001). In addition, no main effect of data type was observed in the combined conditions **B_fwd_S** (PA: F_(1,14)_ = 0.0; p = .96; rTPA: F_(1,14)_ = 0.0; p = .89; RT: F_(1,14)_ = 1.5; p = .25; MD: F_(1,14)_ = 0.5; p = .48) and **B_fwd_S_fwd_** (PA: F_(1,14)_ = 0.0; p = 1.0; rTPA: F_(1,14)_ = 3.1; p = .10; RT: F_(1,14)_ = 1.1; p = .30; MD: F_(1,14)_ = 0.2; p = .67). Finally, no interaction data type x angle of tilt appeared for **B_fwd_S** (PA: F_(2,28)_ = 1.1; p = .34; rTPA: F_(2,28)_ = 1.1; p = .35; RT: F_(2,28)_ = 2.9; p = .07; MD: F_(2,28)_ = 0.2; p = .84) or **B_fwd_S_fwd_** (PA: F_(2,28)_ = 1.9; p = .17 rTPA: F_(2,28)_ = 0.8; p = .46; RT: F_(2,28)_ = 0.9; p = .42; MD: F_(2,28)_ = 0.8; p = .45). In summary, the kinematic variables predicted by the gravity-centered model fitted with the observed data for both combined conditions **B_fwd_S** and **B_fwd_S_fwd_**.

## Discussion

This experiment was designed to investigate whether slow pitch tilts of the body and/or the visual scene influence the organization of arm pointing movements toward a visual target. Overall, body and/or visual scene tilts both induced final errors as compared to non-tilted situations. Tilting the visual scene alone yielded final pointing errors depending on the direction of the visual scene. Tilting the body forward with a scene kept parallel relative to the observer yielded undershoots (negative errors) with respect to the non-tilted situations. The effect of actual body tilt on movement execution could be observed early (i.e., at peak acceleration), thus reflecting changes in motor planning, and appeared to be independent of the scene orientation. When defined in a body-centered reference frame, combined conditions including body and visual scene tilts also induced final errors, which were differently related to the final errors observed in the corresponding single body or scene tilt conditions. The final errors issued from forward body tilt associated to backward scene tilt corresponded to the additive combination (i.e., unweighted sum) of the final errors in the related single stimulations. In contrast, the final errors observed with forward body tilt associated to forward scene tilt appeared close to those observed during single body tilt. Data modeling based on a gravity-centered reference frame appears to offer a unifying explanation for the whole data since predicted final errors never differed from observed ones. These points will be further discussed in the following sections.

### Scene tilt affected perceived target location and sensed gravity orientation

Final errors appeared to vary as a function of the direction of visual scene tilt, with overshoots for forward visual scene tilt and undershoots for backward visual scene tilt. This result may be interpreted as an altered estimation of target location caused by the scene tilt rather than by self- or target-motion perception when considering the characteristics of the visual stimulation (i.e., slow pitch tilt with reduced visual motion). Indeed, several authors suggested that small central field of view and low velocity rotation do not induce vection [31], [37]. In line with our interpretation, a pitch-tilted scene has been found to bias the perceived target elevation [4], [38], [39]. Matin and Fox [4] indeed showed that when the perceived eye level is lowered by a downward (i.e., top forward) room tilt, objects located at physical eye level appear to be higher. Conversely, when the perceived eye level is elevated by an upward (i.e., top backward) room tilt, objects located at physical eye level appear lower. As a consequence, goal-directed movements performed here without body tilt would be altered in the direction of the misperceived target location, i.e., overshoots for forward tilts inducing subjective target elevation and undershoots for backward tilts inducing subjective target lowering. The increase in final errors between 6 and 12 deg, for both visual scene rotations, are consistent with Ballinger unpublished study [5] in which absolute final errors were of 1.6 and 2.2 deg relative to baseline for 7.5 and 15 deg of room pitch tilt, respectively. In addition, the relative higher effect of the backward visual scene rotation as compared to the forward visual scene rotation (i.e., significant pointing error only at 12 deg compared to 12 deg and 18 deg, respectively) is in line with Welch and Post results [1] which showed higher effect of backward as compared to forward 20 deg pitch tilt of a room (≈2.3 deg vs. ≈1.2 deg relative to baseline, respectively).

In addition to the previous interpretation, we suggest that the estimation of gravity orientation relative to the body may have been altered and thus, may have affected arm pointing kinematics but might involve reduced final errors [1]. In a visual changing environment, target position coding relative to an external reference such as gravity would seem an efficient strategy due to its invariant properties [40]–[43]. In addition, Welch and Post [1] previously suggested that the target was not purely coded relative to an egocentric reference frame (i.e., coding relative to the body) as, in their study, pointing errors were lower than perceptual errors associated to eye level estimation (corresponding to a pure egocentric target localization). Here, we found that some kinematic changes associated to visual scene tilts were expressed in a similar way during actual forward body tilt (i.e., non-significant effects of condition and condition x angle). This supported a, at least partial, external target coding implying here that the target location to an altered estimate of gravity orientation, as suggested by Welch and Post [1]. Specifically, movement patterns were found to be more ‘cautious’ when the scene and/or the body were tilted alone, compared to non-tilted situations (longer reaction time, longer movement duration and shorter time-to-peak acceleration relative to movement duration). These findings are in line with those of Gaveau and Papaxanthis [35] who found that slower upward arm pointing movements (i.e., longer movement duration) exhibited a decreased duration of the first movement phase. (i.e., shorter time-to-peak acceleration relative to movement duration) compared to faster movements. Here, we observed a comparable change in motor organization when subjects faced body and/or scene tilts in which arm movement duration was longer. Specifically, the duration of the last movement phase was increased when subjects and/or visual scene were no longer aligned with gravity, which might allow for a greater online control during movement execution. According to several authors [27], [44], [45], this result suggests that subjects would encounter difficulties in integrating the direction of gravity relative to the body in the motor command during a complex visuopostural situation (i.e., when visual and/or body orientation was no longer aligned with gravitational vertical). This complex visuopostural situation could also be linked to the fact that none of our experimental conditions induced fully coherent multisensory stimulation as the visual scene was not rotated around the same axis as the body. Such difficulties in integrating the direction of gravity relative to the body has also been suggested by Welch and Post [1] but this hypothesis was not at that time fully supported by specific kinematic observations.

### Specific changes in movement kinematics due to actual body tilt

**Figure 10:**
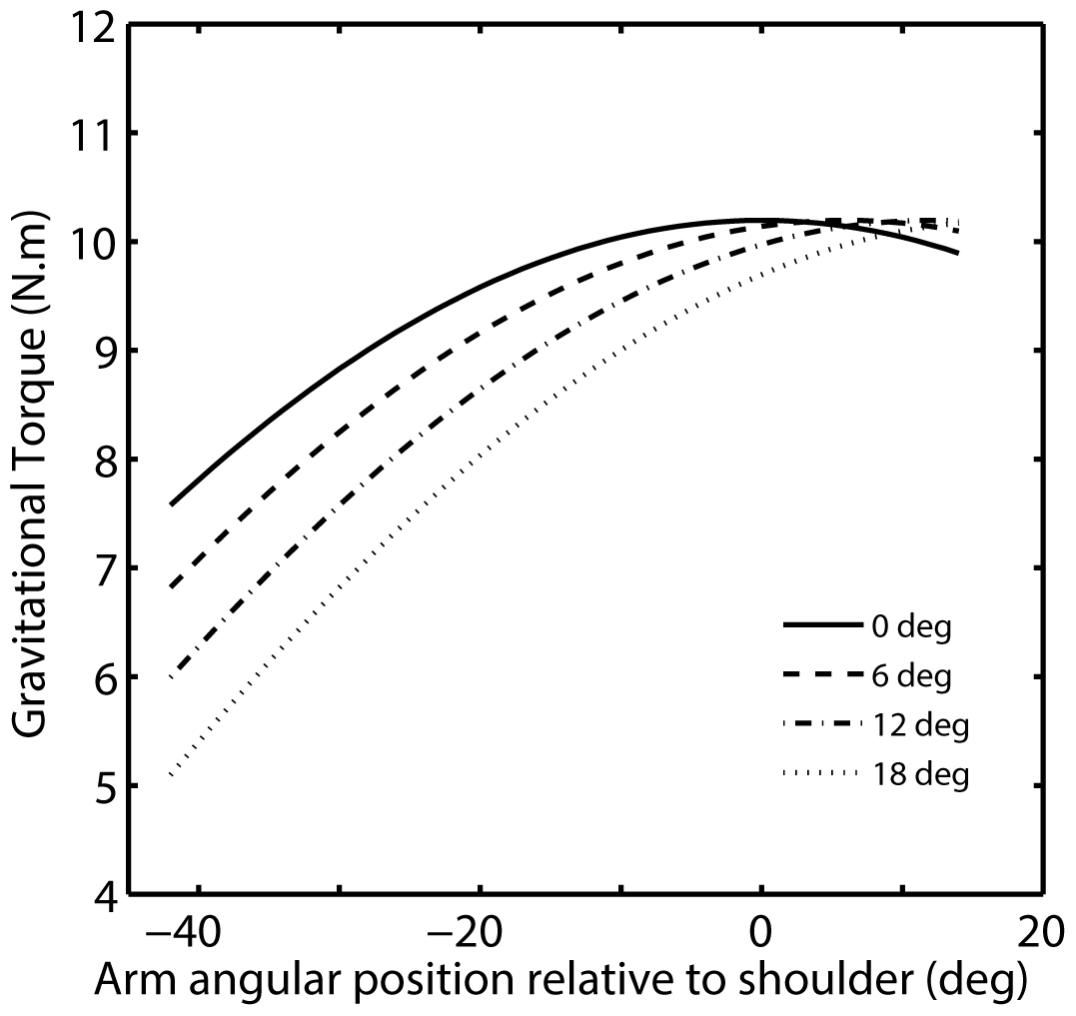
Theoretical gravitational torque at the centre of mass of the arm for each body tilt (0 to 18 deg) as a function of arm angular position relative to the shoulder horizon. Torque was provided from the arm starting position (mean arm position relative to the shoulder = -42 deg) to the final required arm position at eye level (mean arm position relative to the shoulder = 14 deg). Values correspond to an average subject of 70 kg with a 0.35 m upperarm, a 0.30 m forearm, a 0.20 m hand and eye-shoulder distance of 0.21 m.

We found that forward body tilt associated to a scene kept parallel relative to the observer induced modifications of the motor plan, as reflected by the analysis of peak acceleration, and produced final undershoots with respect to the non-tilted baseline. Noticeably, body tilt induced final pointing errors whose magnitude was comparable to that observed with scene tilt. Therefore, one may expect that such pointing modifications could be linked to a similar process based on the combination of cues related to the visual scene and/or the body orientation relative to the vertical. Geometrically speaking, tilting the body did not require updating the egocentric location of a target that was always presented at Head-Referenced Eye level. However, when an observer has to point toward the intended target, he has to take into account the changes of gravity constraints acting on the body and, particularly, on the arm (i.e., gravity-centered coding is required). We suggest that modifications of arm pointing movements during forward body tilt may not be the consequence of target localization errors, as previous results showed that roll body tilt does not induce changes in the accuracy of horizontal target localization [46]. Rather, we argue for an inadequate sensorimotor implementation of the estimated gravitational influence in arm motor planning and control, namely an overestimation of the biomechanical facilitatory influence of gravity upon arm elevation on arm pointing movement. Indeed, when subjects are tilted forward, gravity facilitates the arm shoulder elevation as the gravitational torque to overcome is, on average, smaller (see Figure 10). As a consequence, the required force to rotate the arm toward the target located at HREL was lower, particularly at movement onset. Had subjects not take into account the change of gravity direction relative to their arm prior to or during movement execution and executed the same arm motor command when tilted as when non-tilted, they would have overshot the target (positive errors relative to the baseline). On the contrary, our data showed that body tilt induced final undershoots (negative errors relative to the baseline). This result is rather consistent with previous studies reporting small -however non significant- undershoots during forward body tilt without visual scene [2], [10]. For instance, Bourdin et al. [10] reported final pointing errors of −1.04, −0.39 and −0.98 deg for body tilt at 2, 4 and 8 deg, respectively.

We suggest that final pointing errors observed during slow body tilt may not be considered as simple biomechanical consequences of body tilt. In line with this claim, several studies support the idea of a prior integration of predicted gravitational effects on arm motor command [19]–[21], [35], [36], [47]–[49]. Here we also found that arm movement control was modified at an early stage since body tilt, whatever the scene orientation, induced an early modification of movement pattern with a lower PA compared to when the body was not tilted. Given that gravitational torque applied on the arm when tilted would increase the PA, our findings support the idea of a predictive control of the arm movement taking into account the consequences of gravity on the arm [22]. This hypothesis is based on studies that have already showed that early movement features (e.g., PA, rTPA) reflect motor planning [19], [27], [28]. However, this predictive control may not be fully adapted as, at movement endpoint, we still found final undershoots when the body was tilted forward. Final undershoots associated to lower PA have been also found in microgravity [49], [50], also suggesting a prior overcompensation for the biomechanical consequences of weightlessness on the arm. Overcompensation might be the optimal solution when subjects encounter difficulty integrating gravity, as undershooting can be viewed as functional since movement length, energy and time are all minimized.

### Combination of errors induced by visual scene and body tilt

Overall, we found final undershoots for both combined conditions regardless of the scene tilt direction. Considering our conditions in a body-centered reference frame, we found that when the body was tilted forward and the scene was tilted backward, final pointing errors appeared as an additive combination (i.e., unweighted sum) of the errors observed for single body and scene tilt alone. On the other hand, when both the body and the scene were tilted forward, final pointing errors could not be fully accounted for by this model. We suggest that this difference of combination could be linked to the absolute orientation of the visual scene relative to gravitational vertical. We extended the latter hypothesis by considering that the control of arm movement could have been performed by encoding the body and visual cues in a gravity-centered reference frame. This hypothesis is supported by the absence of difference between all predicted and observed data (final error and kinematic parameters) when conditions are defined relative to gravity.

The link between final errors and gravity can be discussed first when considering experimental conditions in a body-centered reference frame. Indeed, final errors differed between combined conditions **B_fwd_S_fwd_** and **B_fwd_S_bwd_** as a function of the direction of rotation of the visual scene relative to the observer. Nonetheless in these conditions, the visual scene orientation also differed relative to gravitational vertical. While the combined condition including forward body and scene tilts induced an increased deviation of the scene orientation relative to gravity (see Figure 2), the combination of forward body tilt and backward scene tilt kept the visual scene always parallel to gravitational vertical. Previous studies already showed a substantial influence of ‘visual gravity’ in spatial orientation tasks and sensorimotor tasks [12], [27]. Specifically, Sciutti et al. [27] recently showed that visual vertical feedback influenced the planning of horizontal pointing movements whereas horizontal visual feedback did not affect the planning of vertical pointing movements. According to Le Seac'h & McIntyre [12], motor commands need to anticipate gravity consequences on motor execution, and gravitational vertical is taken into account through multiple sensory cues, notably visual. Gravitational vertical may have a particular status compared to the other directions [51]. Indeed, several studies suggested that it would be integrated as an internal model [20], [22]. In one condition of the present experiment, the fact that the visual scene remained oriented at gravitational vertical could increase its relevance. Conversely, when the visual scene was no longer aligned with gravity, the weight of its associated spatial cues might have substantially decreased in favour of body-related cues. However, dominance of body-related cues when the visual scene is not aligned with gravity, does not automatically mean that the weight of visual cues is decreased when other body-related cues are available. Indeed, we observed an influence of single visual scene rotation while static body orientation cues remained available. Therefore, combination of spatial cues might also depend on the nature of body-related cues: static (i.e., unchanged body orientation) versus dynamic (i.e., actual –even slow– body rotation). Overall, we argue that the absolute orientation of the scene appears determinant in the combination process.

While absolute vertical may play a major role in the control of pointing movements considering a body-centered reference frame, one may further hypothesize that the absolute vertical could constitute the reference for encoding visual and non-visual orientation cues. In such a gravity-centered reference frame, the condition including forward body tilt and backward visual scene tilt relative to the observer can be considered a single condition because it provides a stable visual reference in space. In parallel, the combined conditions in this gravity-centered reference frame included a perturbation of the visual scene orientation relative to gravity as well as body orientation (i.e., **B_fwd_S** and **B_fwd_S_fwd_** conditions). Our results indicate that a linear combination of data in the single conditions can account for the data in both of these combined conditions, when we consider final errors as well as all tested kinematic variables. Overall these results support the idea that gravity is an invariant reference for the planning and control of pointing movement. Gravity-centered coding would here enable a more reliable reference frame than body-centered coding, mainly because both the visual and body orientation were modified in our experiment. This hypothesis is supported by the study of Burns and Blohm [45] showing that the encoding of pointing movement characteristics relies more on external references than egocentric ones when the head is tilted. Gravity-centered coding hence would provide a stable reference frame for movement control [43], [52], an idea consistent with the report that in the absence of gravity, goal-directed movements become inaccurate (for review see [53]). Recently, Tagliabue et al. [44] added that there is no prior given to the egocentric reference frame when performing a sensorimotor task. With the principles of the Maximum Likelihood Estimation [54], these authors [44] demonstrated that the CNS tends to maximise the weight of spatial coordinates that minimize the output variability. In the present experiment, one might expect that body-related and visual cues relative to gravity led to less variable arm movements, a hypothesis which needs to be tested in further experiments.

## Conclusion

We showed that pointing toward a target during slow body and visual scene tilt provides a way to investigate combination rules of spatial cues involved in sensorimotor control. Our results suggest that the gravity plays a crucial role for the planning and control of arm pointing movements. The CNS may use gravity, thanks to its invariant properties, as a reference for the combination of visual and non-visual cues. The selected form of combination process expressed in the control of arm pointing movements may then arise from the spatial context mediated by the available cues.

## Appendix

The influence of pointing succession was investigated through 5 condition×4 angle of tilt×6 pointing succession ANOVAs performed on final position, PA, rTPA, RT and MD. First, a main effect of pointing succession was found for PA, RT and MD (see Table S1).

Second, the analyses revealed that the interaction angle of tilt x pointing succession was significant for final position, PA, RT and MD (Table S1). Overall, as presented in Figure S1, the evolution across pointing succession did not follow a clear pattern. Indeed, the analysis of final position, RT and MD did not show a global increase or decrease across pointing for tilted compared to non-tilted situations. These results suggested that the continuous rotation, used in our protocol, was not the cause of the pointing succession effect. We rather suggest that the pointing succession effect, due to the repetitions of pointing movements, differed in tilted and non-tilted conditions because of differences in visuo-postural constraints. For PA and MD, the interaction angle of tilt x pointing succession mainly suggested that the first pointing movement (P1) differed from the subsequent ones, since from P2 to P6 the pattern of pointing was similar for each angle of tilt (Fig. S1b and S1d). When comparing P1 to the other pointing movements, MD tended to be longer and PA smaller when tilted, probably reflecting the exposure to a new perturbation (see also section *Kinematic parameters*). The presence of a novel perturbation (i.e., visual and/or body rotation) could also explain why the RT was reduced across trials at a slower rate in tilted conditions as compared to 0 deg (see Figure S1c). Finally, the results showed that contrary to non-tilted situations, the tilted situations did not induce a high modulation of the final positions across the successive pointing movements (see Figure S1a). We argued that the regulation of final position over the successive pointing movements at 0 deg was no longer possible when the situation was perturbed (i.e., visual and/or body rotation).

Most importantly, the interaction condition x pointing succession and the interaction condition x angle of tilt x pointing succession were not significant for all variables (Table S1). These results indicate that the described statistical effects did not affect the interaction between the angle of tilt and condition, which remains the primary interest of the study.

Finally, an interaction condition x angle of tilt was found for final position and PA. For final position, post-hoc results revealed that in the condition **B_fwd_S_bwd_**, 0 deg differed from 6, 12 and 18 deg (p<.001 for all comparisons), and in the condition **S_bwd_**, 0 deg differed from 12 and 18 deg (p<.01 and p<.05, respectively). Planned comparisons showed statistical differences in final position between 0 deg and tilted situations for **S_bwd_** (p<.05), **B_fwd_S** (p<.05), **B_fwd_S_bwd_** (p<.001) but not for **S_fwd_** (p = .13) and **B_fwd_S_fwd_** (p = .42). Planned comparisons showed statistical differences in PA between 0 deg and tilted situations for **S_bwd_** (p<.05), **B_fwd_S** (p<.01), **B_fwd_S_fwd_** (p<.01) and **B_fwd_S_bwd_** but not for **S_fwd_** (p = .75). Overall, these latter results were similar to those presented in the main manuscript (See sections *Final Accuracy* and *Movement kinematics*) were we averaged the values obtained from 6 pointing movements x 3 repetitions.

## Supporting Information

Figure S1 **Interaction pointing succession x angle of tilt for final position (a), PA (b), RT (c) and MD (d).** Vertical bars denote positive standard errors. Statistical differences between pointing movements for a given angle of tilt are provided in Table S2.
(EPS)

Table S1 **Statistical results of the 5 condition x 4 angle of tilt x 6 pointing succession ANOVAs performed on final position, PA, rTPA, RT and MD.** Significant effects are presented in bold. *: p<.05; ‡: p<.01; †: p<.001.
(EPS)

Table S2 **Post-hoc results of the significant angle of tilt x pointing succession interactions which were revealed on final position, PA, rTPA, RT and MD.** This table provides statistical differences between pointing movements for a given angle of tilt. n.s: non-significant, *: p<.05; ‡: p<.01; †: p<.001.
(EPS)
